# Lithium bis­(2-methyl­lactato)borate monohydrate

**DOI:** 10.1107/S1600536812017540

**Published:** 2012-05-12

**Authors:** Joshua L. Allen, Elie Paillard, Paul D. Boyle, Wesley A. Henderson

**Affiliations:** a Ionic Liquids and Electrolytes for Energy Technologies (ILEET) Laboratory, Dept. of Chemical and Biomolecular Engineering, North Carolina State University, 911 Partners Way, Raleigh, NC 27695, USA; b X-ray Structural Facility, Dept. of Chemistry, North Carolina State University, 2620 Yarbrough Drive, Raleigh, NC 27695, USA

## Abstract

The title compound {systematic name: poly[[aqua­lithium]-μ-3,3,8,8-tetra­methyl-1,4,6,9-tetra­oxa-5λ^4^-borataspiro­[4.4]nonane-2,7-dione]}, [Li(C_8_H_12_BO_6_)(H_2_O)]_*n*_ (LiBMLB), forms a 12-membered macrocycle, which lies across a crystallographic inversion center. The lithium cations are pseudo-tetra­hedrally coordinated by three methyl­lactate ligands and a water mol­ecule. The asymmetric units couple across crystallographic inversion centers, forming the 12-membered macrocycles. These macrocycles, in turn, cross-link through the Li^+^ cations, forming an infinite polymeric structure in two dimensions parallel to (101).

## Related literature

For the synthesis and purification of HBMLB [BMLB is bis­(2-methyl­lactato)borate], see: Lamande *et al.* (1987 ▶). For the synthesis and properties of LiBMLB and BMLB^−^-based ionic liquids, see: Xu *et al.* (2003 ▶). For crystallographic data of similar lithium salts, see: Zavalij *et al.* (2004 ▶); Allen *et al.* (2011 ▶).
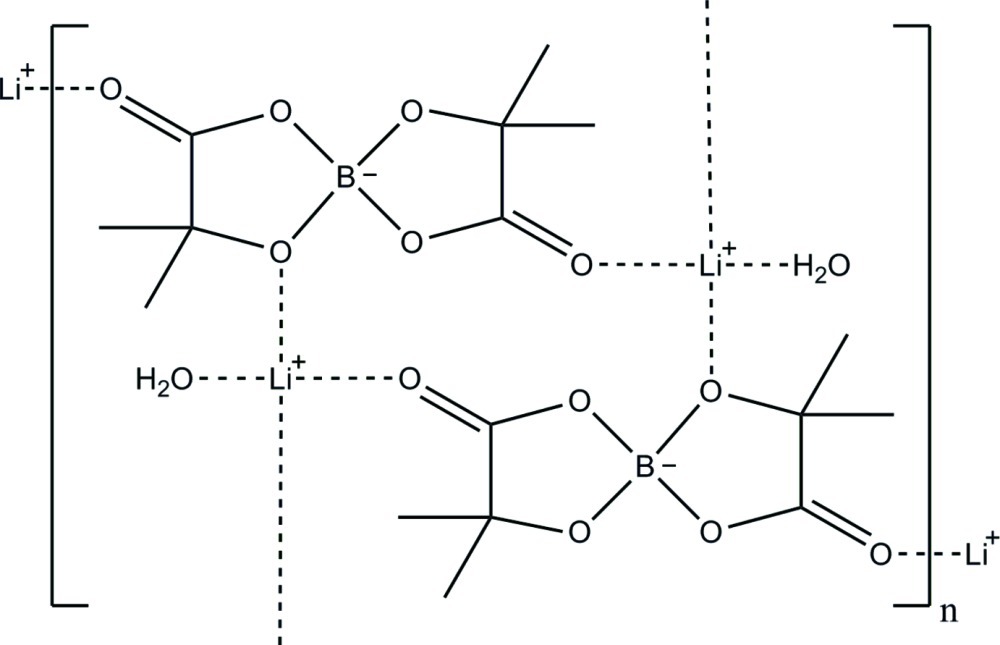


## Experimental

### 

#### Crystal data

[Li(C_8_H_12_BO_6_)(H_2_O)]
*M* *_r_* = 239.94
Orthorhombic, 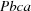

*a* = 12.7034 (4) Å
*b* = 11.3939 (4) Å
*c* = 15.8258 (5) Å
*V* = 2290.65 (13) Å^3^
*Z* = 8
Mo *K*α radiation
μ = 0.12 mm^−1^
*T* = 110 K
0.34 × 0.23 × 0.18 mm

#### Data collection

Bruker–Nonius Kappa X8 APEXII diffractometer
Absorption correction: multi-scan (*SADABS*; Bruker, 2007 ▶) *T*_min_ = 0.961, *T*_max_ = 0.979
97648 measured reflections
5663 independent reflections
4436 reflections with *I* > 2σ(*I*)
*R*_int_ = 0.037

#### Refinement

*R*[*F* ^2^ > 2σ(*F* ^2^)] = 0.036
*wR*(*F* ^2^) = 0.098
*S* = 1.05
5663 reflections
210 parameters
All H-atom parameters refined
Δρ_max_ = 0.51 e Å^−3^
Δρ_min_ = −0.26 e Å^−3^

### 

Data collection: *APEX2* (Bruker, 2007 ▶); cell refinement: *SAINT* (Bruker, 2007 ▶); data reduction: *SAINT*; program(s) used to solve structure: *SIR92* (Altomare *et al.*, 1994 ▶); program(s) used to refine structure: *XL* (Sheldrick, 2008 ▶); molecular graphics: *ORTEP-3* (Farrugia, 1997 ▶); software used to prepare material for publication: *cif2tables.py* (Boyle, 2008 ▶).

## Supplementary Material

Crystal structure: contains datablock(s) I, global. DOI: 10.1107/S1600536812017540/vn2036sup1.cif

Structure factors: contains datablock(s) I. DOI: 10.1107/S1600536812017540/vn2036Isup2.hkl

Additional supplementary materials:  crystallographic information; 3D view; checkCIF report

## Figures and Tables

**Table 1:** Selected bond lengths (Å)

Li1—O1	1.9725 (13)
Li1—O1*W*	1.9487 (13)
Li1—O3^i^	2.0059 (13)
Li1—O6^ii^	1.9155 (13)

Symmetry codes: (i) 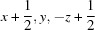; (ii) 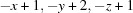.

**Fig. 1.**
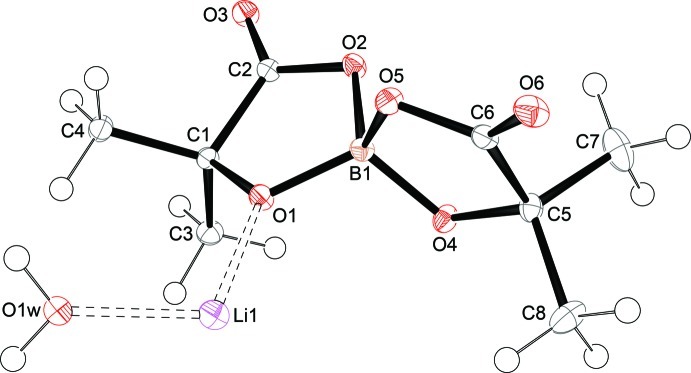
Asymmetric unit of LiBMLB-H2O showing naming and numbering scheme. Thermal ellipsoids are at 50% probability (Li-purple, O-red, B-tan, C-grey).

**Fig. 2.**
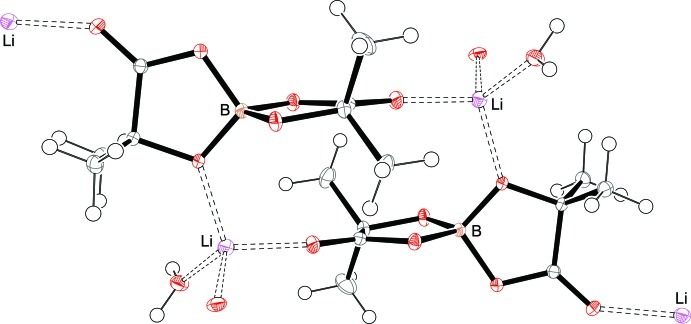
A 12-membered macrocycle formed from two LiBMLB-H2O units. Thermal ellipsoids are at 50% probability (Li-purple, O-red, B-tan, C-grey).

**Fig. 3.**
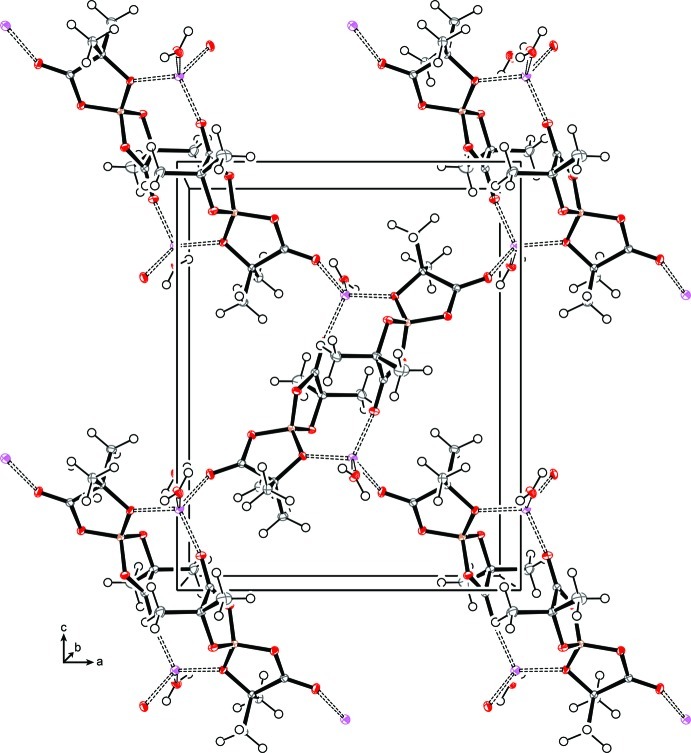
A portion of the unit cell of [LiBMLB-H2O]n. Thermal ellipsoids are at 50% probability (Li-purple, O-red, B-tan, C-grey).

## supplementary crystallographic information

### Comment

Various lithium salts for lithium-ion batteries have been proposed in recent years either as alternatives to the commonly used lithium hexafluorophosphate (LiPF_6_) or as electrolyte additives. Of these salts, lithium bis(oxalato)borate [LiBOB] remains one of the most promising (Zavalij *et al.*). The title compound, lithium bis(2-methyllactato)borate [LiBMLB] is based on this structure, differing only by replacing the oxygen of a carbonyl group of each ligand with two methyl groups. Although this salt has previously been synthesized (Lamande *et al.*, Xu *et al.*), the crystal structure and ion coordination have not yet been reported. The structure of the monohydrate solvate of this salt is reported in the present manuscript.

The Li^+^ cation coordination in the title compound is different from what has been previously reported for similar cyclic structures (Allen *et al.*, Zavalij *et al.*). For salts such as LiBOB, the Li^+^ cations are exclusively coordinated by the anion carbonyl oxygen atoms. In the present structure, however, the anion ring pseudo-ether oxygen also participates in the Li^+^ cation coordination (Fig. 1). Thus, each Li^+^ cation is coordinated by two carbonyl oxygen atoms from two BMLB^-^ anions, one ring oxygen from a third BMLB^-^ anion and an oxygen from a single water molecule. The asymmetric unit couples across crystallographic inversion centers to form 12-membered macrocycles (Fig. 2). These macrocycles are cross-linked through the Li^+^ cation coordination, forming the infinite polymeric crystal structure in two dimensions parallel to (101) (Fig. 3).

### Experimental

Lithium bis(2-methyllactato)borate was synthesized by dissolving 2-methyllactic acid, boric acid and lithium carbonate (mole ratio 4:2:1) in water. The aqueous solution was allowed to slowly evaporate, forming colorless crystals suitable for X-ray analysis.

### Refinement

The hydrogen atom positional and isotropic displacement parameters were included in the refinement.

### Crystal data

**Table:**

[Li(C_8_H_12_BO_6_)(H_2_O)]	*F*(000) = 1008
*M**_r_* = 239.94	*D*_x_ = 1.392 Mg m^−^^3^
Orthorhombic, *P**b**c**a*	Mo *K*α radiation, λ = 0.71073 Å
Hall symbol: -P 2ac 2ab	Cell parameters from 9969 reflections
*a* = 12.7034 (4) Å	θ = 2.7–35.0°
*b* = 11.3939 (4) Å	µ = 0.12 mm^−^^1^
*c* = 15.8258 (5) Å	*T* = 110 K
*V* = 2290.65 (13) Å^3^	Prism, colourless
*Z* = 8	0.34 × 0.23 × 0.18 mm

### Data collection

**Table:**

Bruker–Nonius Kappa X8 APEXII diffractometer	5663 independent reflections
Radiation source: fine-focus sealed tube	4436 reflections with *I* > 2σ(*I*)
Graphite monochromator	*R*_int_ = 0.037
ω and φ scans	θ_max_ = 37.4°, θ_min_ = 2.6°
Absorption correction: multi-scan (*SADABS*; Bruker, 2007)	*h* = −21→21
*T*_min_ = 0.961, *T*_max_ = 0.979	*k* = −19→19
97648 measured reflections	*l* = −24→26

### Refinement

**Table:**

Refinement on *F*^2^	Primary atom site location: structure-invariant direct methods
Least-squares matrix: full	Secondary atom site location: difference Fourier map
*R*[*F*^2^ > 2σ(*F*^2^)] = 0.036	Hydrogen site location: inferred from neighbouring sites
*wR*(*F*^2^) = 0.098	All H-atom parameters refined
*S* = 1.05	*w* = 1/[σ^2^(*F*_o_^2^) + (0.0519*P*)^2^ + 0.3146*P*] where *P* = (*F*_o_^2^ + 2*F*_c_^2^)/3
5663 reflections	(Δ/σ)_max_ = 0.001
210 parameters	Δρ_max_ = 0.51 e Å^−^^3^
0 restraints	Δρ_min_ = −0.26 e Å^−^^3^

### Special details

**Table:**

Geometry. All e.s.d.'s (except the e.s.d. in the dihedral angle between two l.s. planes) are estimated using the full covariance matrix. The cell e.s.d.'s are taken into account individually in the estimation of e.s.d.'s in distances, angles and torsion angles; correlations between e.s.d.'s in cell parameters are only used when they are defined by crystal symmetry. An approximate (isotropic) treatment of cell e.s.d.'s is used for estimating e.s.d.'s involving l.s. planes.
Refinement. Refinement of *F*^2^ against ALL reflections. The weighted *R*-factor *wR* and goodness of fit *S* are based on *F*^2^, conventional *R*-factors *R* are based on *F*, with *F* set to zero for negative *F*^2^. The threshold expression of *F*^2^ > σ(*F*^2^) is used only for calculating *R*-factors(gt) *etc*. and is not relevant to the choice of reflections for refinement. *R*-factors based on *F*^2^ are statistically about twice as large as those based on *F*, and *R*- factors based on ALL data will be even larger.

### Fractional atomic coordinates and isotropic or equivalent isotropic displacement parameters (Å^2^)

**Table:**

	*x*	*y*	*z*	*U*_iso_*/*U*_eq_	
Li1	0.50924 (9)	0.90999 (11)	0.30769 (8)	0.0141 (2)	
O1	0.36589 (3)	0.97594 (4)	0.31383 (3)	0.01110 (9)	
O2	0.21632 (3)	1.07434 (4)	0.36227 (3)	0.01158 (9)	
O3	0.09643 (4)	1.00827 (4)	0.27079 (3)	0.01399 (9)	
O4	0.38947 (4)	1.16989 (4)	0.37533 (3)	0.01216 (9)	
O5	0.34797 (4)	1.01520 (4)	0.46522 (3)	0.01186 (9)	
O6	0.42797 (4)	1.07518 (5)	0.58279 (3)	0.01560 (10)	
B1	0.33286 (5)	1.05967 (6)	0.37751 (4)	0.01016 (11)	
C1	0.28304 (4)	0.95743 (5)	0.25349 (4)	0.00955 (10)	
C2	0.18775 (5)	1.01423 (5)	0.29565 (4)	0.01021 (10)	
C3	0.30791 (5)	1.02291 (6)	0.17184 (4)	0.01333 (11)	
H3A	0.3167 (9)	1.1064 (10)	0.1823 (7)	0.024 (3)*	
H3B	0.2509 (10)	1.0133 (10)	0.1323 (7)	0.023 (3)*	
H3C	0.3744 (9)	0.9899 (9)	0.1465 (7)	0.020 (2)*	
C4	0.26552 (5)	0.82752 (5)	0.23795 (4)	0.01314 (11)	
H4A	0.3291 (9)	0.7933 (10)	0.2120 (7)	0.025 (3)*	
H4B	0.2057 (9)	0.8176 (9)	0.1987 (7)	0.020 (2)*	
H4C	0.2505 (8)	0.7861 (9)	0.2889 (7)	0.019 (2)*	
C5	0.42313 (5)	1.20239 (5)	0.45830 (4)	0.01192 (11)	
C6	0.40175 (5)	1.09179 (5)	0.50964 (4)	0.01110 (11)	
C7	0.35561 (7)	1.30226 (7)	0.49223 (5)	0.02424 (16)	
H7A	0.3687 (10)	1.3751 (11)	0.4564 (8)	0.033 (3)*	
H7B	0.3770 (11)	1.3184 (12)	0.5514 (9)	0.041 (3)*	
H7C	0.2815 (11)	1.2823 (12)	0.4919 (8)	0.036 (3)*	
C8	0.53960 (6)	1.23386 (7)	0.45795 (5)	0.01929 (13)	
H8A	0.5830 (10)	1.1701 (11)	0.4330 (8)	0.032 (3)*	
H8B	0.5636 (9)	1.2482 (10)	0.5166 (8)	0.030 (3)*	
H8C	0.5506 (9)	1.3071 (10)	0.4247 (7)	0.026 (3)*	
O1W	0.51220 (4)	0.74779 (4)	0.26871 (3)	0.01548 (10)	
H1WA	0.5426 (12)	0.7243 (12)	0.2236 (10)	0.046 (4)*	
H1WB	0.4774 (11)	0.6878 (12)	0.2863 (9)	0.041 (3)*	

### Atomic displacement parameters (Å^2^)

**Table:**

	*U*^11^	*U*^22^	*U*^33^	*U*^12^	*U*^13^	*U*^23^
Li1	0.0132 (5)	0.0162 (5)	0.0128 (5)	0.0010 (4)	−0.0010 (4)	0.0001 (4)
O1	0.00863 (17)	0.0148 (2)	0.00990 (19)	0.00106 (14)	−0.00213 (14)	−0.00282 (15)
O2	0.00987 (18)	0.01454 (19)	0.0103 (2)	0.00099 (14)	−0.00098 (14)	−0.00277 (15)
O3	0.00931 (18)	0.0180 (2)	0.0147 (2)	0.00129 (15)	−0.00238 (16)	−0.00252 (17)
O4	0.0154 (2)	0.01258 (19)	0.00853 (18)	−0.00325 (15)	−0.00187 (15)	0.00066 (15)
O5	0.01270 (19)	0.01361 (19)	0.00925 (19)	−0.00208 (15)	−0.00121 (15)	0.00105 (15)
O6	0.0148 (2)	0.0233 (2)	0.0087 (2)	0.00015 (17)	−0.00136 (16)	0.00053 (17)
B1	0.0099 (2)	0.0121 (3)	0.0085 (3)	−0.0002 (2)	−0.0005 (2)	−0.0001 (2)
C1	0.0085 (2)	0.0109 (2)	0.0092 (2)	−0.00022 (17)	−0.00104 (18)	−0.00056 (19)
C2	0.0104 (2)	0.0109 (2)	0.0094 (2)	0.00061 (18)	−0.00024 (18)	0.00053 (18)
C3	0.0150 (3)	0.0149 (3)	0.0101 (2)	−0.0014 (2)	0.0006 (2)	0.0015 (2)
C4	0.0122 (2)	0.0107 (2)	0.0166 (3)	−0.00059 (18)	−0.0002 (2)	−0.0010 (2)
C5	0.0137 (2)	0.0123 (2)	0.0098 (2)	−0.00051 (19)	−0.00179 (19)	−0.00122 (19)
C6	0.0093 (2)	0.0145 (2)	0.0095 (2)	0.00077 (18)	0.00041 (18)	−0.0008 (2)
C7	0.0333 (4)	0.0180 (3)	0.0214 (3)	0.0094 (3)	0.0012 (3)	−0.0049 (3)
C8	0.0167 (3)	0.0213 (3)	0.0198 (3)	−0.0076 (2)	−0.0040 (2)	0.0027 (3)
O1W	0.0147 (2)	0.0146 (2)	0.0172 (2)	−0.00084 (16)	0.00323 (17)	−0.00317 (17)

### Geometric parameters (Å, º)

**Table:**

Li1—O1	1.9725 (13)	C1—C2	1.5263 (8)
Li1—O1W	1.9487 (13)	C3—H3A	0.972 (11)
Li1—O3^i^	2.0059 (13)	C3—H3B	0.963 (12)
Li1—O6^ii^	1.9155 (13)	C3—H3C	1.007 (11)
O1—C1	1.4366 (7)	C4—H4A	0.987 (12)
O1—B1	1.4498 (8)	C4—H4B	0.988 (11)
O2—C2	1.3086 (7)	C4—H4C	0.953 (11)
O2—B1	1.5094 (8)	C5—C8	1.5223 (9)
O3—C2	1.2268 (7)	C5—C7	1.5228 (10)
O3—Li1^iii^	2.0059 (13)	C5—C6	1.5238 (9)
O4—C5	1.4297 (8)	C7—H7A	1.019 (13)
O4—B1	1.4476 (8)	C7—H7B	0.993 (14)
O5—C6	1.3125 (8)	C7—H7C	0.968 (13)
O5—B1	1.4901 (8)	C8—H8A	0.994 (13)
O6—C6	1.2193 (8)	C8—H8B	0.990 (12)
O6—Li1^ii^	1.9155 (13)	C8—H8C	0.997 (12)
C1—C4	1.5169 (8)	O1W—H1WA	0.854 (16)
C1—C3	1.5251 (9)	O1W—H1WB	0.861 (14)
			
O6^ii^—Li1—O1W	111.23 (6)	H3A—C3—H3C	109.7 (9)
O6^ii^—Li1—O1	107.84 (6)	H3B—C3—H3C	109.3 (10)
O1W—Li1—O1	113.25 (6)	C1—C4—H4A	109.4 (7)
O6^ii^—Li1—O3^i^	106.33 (6)	C1—C4—H4B	109.1 (6)
O1W—Li1—O3^i^	108.83 (6)	H4A—C4—H4B	108.8 (9)
O1—Li1—O3^i^	109.12 (6)	C1—C4—H4C	112.0 (6)
C1—O1—B1	110.28 (5)	H4A—C4—H4C	108.7 (9)
C1—O1—Li1	125.99 (5)	H4B—C4—H4C	108.7 (9)
B1—O1—Li1	123.51 (5)	O4—C5—C8	110.39 (5)
C2—O2—B1	110.05 (5)	O4—C5—C7	110.42 (6)
C2—O3—Li1^iii^	138.72 (6)	C8—C5—C7	111.88 (6)
C5—O4—B1	110.58 (5)	O4—C5—C6	102.84 (5)
C6—O5—B1	109.87 (5)	C8—C5—C6	111.71 (5)
C6—O6—Li1^ii^	163.55 (6)	C7—C5—C6	109.24 (6)
O4—B1—O1	114.25 (5)	O6—C6—O5	123.19 (6)
O4—B1—O5	104.66 (5)	O6—C6—C5	125.87 (6)
O1—B1—O5	112.74 (5)	O5—C6—C5	110.92 (5)
O4—B1—O2	112.80 (5)	C5—C7—H7A	108.7 (7)
O1—B1—O2	104.22 (5)	C5—C7—H7B	108.4 (8)
O5—B1—O2	108.23 (5)	H7A—C7—H7B	109.3 (11)
O1—C1—C4	111.01 (5)	C5—C7—H7C	111.7 (8)
O1—C1—C3	109.85 (5)	H7A—C7—H7C	110.3 (11)
C4—C1—C3	111.74 (5)	H7B—C7—H7C	108.4 (11)
O1—C1—C2	103.20 (5)	C5—C8—H8A	111.6 (7)
C4—C1—C2	111.60 (5)	C5—C8—H8B	109.5 (7)
C3—C1—C2	109.10 (5)	H8A—C8—H8B	108.8 (10)
O3—C2—O2	123.33 (6)	C5—C8—H8C	109.6 (7)
O3—C2—C1	125.90 (6)	H8A—C8—H8C	109.0 (10)
O2—C2—C1	110.74 (5)	H8B—C8—H8C	108.3 (9)
C1—C3—H3A	111.0 (7)	Li1—O1W—H1WA	124.8 (9)
C1—C3—H3B	109.8 (7)	Li1—O1W—H1WB	129.9 (9)
H3A—C3—H3B	107.9 (9)	H1WA—O1W—H1WB	104.7 (13)
C1—C3—H3C	109.2 (6)		
			
O6^ii^—Li1—O1—C1	−163.76 (5)	B1—O1—C1—C2	−12.51 (6)
O1W—Li1—O1—C1	−40.24 (9)	Li1—O1—C1—C2	172.64 (6)
O3^i^—Li1—O1—C1	81.15 (8)	Li1^iii^—O3—C2—O2	−164.56 (7)
O6^ii^—Li1—O1—B1	22.04 (9)	Li1^iii^—O3—C2—C1	17.31 (12)
O1W—Li1—O1—B1	145.56 (6)	B1—O2—C2—O3	177.79 (6)
O3^i^—Li1—O1—B1	−93.05 (7)	B1—O2—C2—C1	−3.83 (7)
C5—O4—B1—O1	−132.62 (5)	O1—C1—C2—O3	−171.56 (6)
C5—O4—B1—O5	−8.84 (6)	C4—C1—C2—O3	−52.29 (8)
C5—O4—B1—O2	108.60 (6)	C3—C1—C2—O3	71.68 (8)
C1—O1—B1—O4	−112.92 (6)	O1—C1—C2—O2	10.11 (6)
Li1—O1—B1—O4	62.07 (8)	C4—C1—C2—O2	129.38 (5)
C1—O1—B1—O5	127.75 (5)	C3—C1—C2—O2	−106.65 (6)
Li1—O1—B1—O5	−57.25 (8)	B1—O4—C5—C8	130.06 (6)
C1—O1—B1—O2	10.61 (6)	B1—O4—C5—C7	−105.70 (6)
Li1—O1—B1—O2	−174.39 (5)	B1—O4—C5—C6	10.76 (6)
C6—O5—B1—O4	2.75 (6)	Li1^ii^—O6—C6—O5	172.88 (18)
C6—O5—B1—O1	127.49 (5)	Li1^ii^—O6—C6—C5	−8.8 (2)
C6—O5—B1—O2	−117.77 (5)	B1—O5—C6—O6	−177.52 (6)
C2—O2—B1—O4	120.53 (5)	B1—O5—C6—C5	3.98 (7)
C2—O2—B1—O1	−3.95 (6)	O4—C5—C6—O6	172.41 (6)
C2—O2—B1—O5	−124.16 (5)	C8—C5—C6—O6	54.03 (8)
B1—O1—C1—C4	−132.19 (5)	C7—C5—C6—O6	−70.29 (8)
Li1—O1—C1—C4	52.96 (8)	O4—C5—C6—O5	−9.14 (6)
B1—O1—C1—C3	103.72 (6)	C8—C5—C6—O5	−127.51 (6)
Li1—O1—C1—C3	−71.13 (7)	C7—C5—C6—O5	108.16 (6)

Symmetry codes: (i) *x*+1/2, *y*, −*z*+1/2; (ii) −*x*+1, −*y*+2, −*z*+1; (iii) *x*−1/2, *y*, −*z*+1/2.
